# The prevalence of and factors associated with antenatal depression among all pregnant women first attending antenatal care: a cross-sectional study in a comprehensive teaching hospital

**DOI:** 10.1186/s12884-021-04090-z

**Published:** 2021-10-26

**Authors:** Jiamei Guo, Anhai Zheng, Jinglan He, Ming Ai, Yao Gan, Qi Zhang, Lulu Chen, Sisi Liang, Xiaoyu Yu, Li Kuang

**Affiliations:** 1 Department of Psychiatry, The First Affiliated Hospital of Chongqing Medical University, Chongqing, 400016 People’s Republic of China; 2 Department of Obstetrics, The First Affiliated Hospital of Chongqing Medical University, Chongqing, 400016 People’s Republic of China

**Keywords:** Antenatal depression, Prevalence, Associated factors, Online psychological assessment

## Abstract

**Background:**

Antenatal depression has become a common and serious problem, significantly affecting maternal and fetal health. However, evaluation and intervention methods for pregnant women in obstetric clinics are inadequate. This study aimed to determine the prevalence of and risk factors for depression among all pregnant women at their first attending antenatal care in the obstetrics clinic, a comprehensive teaching hospital, southwest of China.

**Methods:**

From June to December 2019, 5780 pregnant women completed online psychological assessments, and data from 5728 of the women were analyzed. The women were categorized into two groups according to the presence or absence of depression. Depression was assessed by the Patient Health Questionnaire-9 (PHQ-9), with a cutoff point of 10 for depression. Anxiety and somatic symptoms were measured by the Generalized Anxiety Disorder-7 (GAD-7) and Patient Health Questionnaire-15 (PHQ-15), respectively. Univariate analysis and binary logistic regression analysis were used to determine the association among antenatal depression, anxiety, somatic symptoms and participants’ characteristics.

**Results:**

The prevalence of antenatal depression among all the pregnant women at their first attending antenatal care was 16.3%, higher in the first trimester (18.1%). Anxiety symptoms (Mild anxiety AOR = 2.937; 95% CI: 2.448–3.524) and somatic symptoms (Mild somatic symptoms AOR = 3.938; 95% CI: 2.888–3.368) were major risk factors for antenatal depression among women and the risk increased more with the anxiety level or somatic symptoms level. Gestational weeks (second trimester AOR = 0.611; 95% CI: 0.483–0.773; third trimester AOR = 0.337; 95% CI: 0.228–0.498) and urban residence (AOR = 0.786; 95% CI: 0.652–0.947) were protective factors for antenatal depression among women.

**Conclusions:**

About one in six pregnant women would experience depression, and special attention should be paid to some risk factors (i.e., early pregnancy, anxiety symptoms, somatic symptoms, rural residence). Online psychological assessments might be a time-saving and convenient screening method for pregnant women in obstetric clinics.

## Introduction

Depression has become a common public mental health problem, which would rank first in the world disease burden by 2030 [1] . This problem is seriously affecting the mental health of the Chinese people [2]. Numerous epidemiological studies have shown that women are more susceptible to depression than men, with an approximately 2-fold greater risk [3]. Childbearing age is the highest-risk period for depression among females due to a mixture of hormonal changes and a series of psychosocial factors [4] and depression is more common in pregnant than non-pregnant women. A recently published meta-analysis reported that 16.3% of pregnant women had depression during pregnancy (participants mostly from developed countries) [5]. In low- and middle-income countries, such as Africa, the prevalence of antepartum depression was higher, up to 26.3% [6]. Risk factors of antenatal depression are multifactorial including age of pregnant women, marital status, income, education, occupation, unplanned pregnancy, history of the previous mental disorder, complication during pregnancy, conflicts, social support, number of pregnancy, history of fetal loss, and relationship with husband [6, 7].

Antenatal depression might have a range ogf adverse effects on mothers, infants and families, including postpartum depression [8], pregnancy complications [9], preterm birth [10], low birth weight [11], infant mental development [12], and a higher risk of developing mental disorders in adolescent offspring [13]. Hence, the US Preventive Services Task Force (USPSTF) proposed the recommendation of screening for perinatal depression and anxiety and providing counselling interventions in time for public health [14]. In fact, antepartum depression has been often overlooked by clinical doctors and screening strategies and interventions are scarce. So it is necessary to carry out research on the status of, screening for and intervention in prenatal depression.

Although there have been some studies on the prevalence of and risk factors for antenatal depression in China [15–17], little is known about the status of depression among all pregnant women in obstetrics clinics. To better understand the current status of antenatal depression in China, a maternal MDT (multidisciplinary team) composed of psychiatrists, obstetricians and psychological counsellors was set up in May 2019 in our hospital, and an online screening questionnaire was developed to assess the mental health of all pregnant women in obstetric clinics. The depression was assessed by the Patient Health Questionnaire-9 (PHQ-9) which contains all of the nine symptoms required for DSM-5 diagnosis of major depression. And it is the most commonly used tool for screening for depression in primary care [18], with good reliability and validity among pregnant populations. The Generalized Anxiety Disorder-7(GAD-7) and the Patient Health Questionnaire-15(PHQ-15) were used to assess participants’ anxiety and somatic symptoms. To our knowledge, this was the first study to estimate the burden of depression among all pregnant women at their fist attending antenatal care in obstetrics outpatient department. And, several potential factors associated with prenatal depression were also investigated. The results of our study might contribute to provide valuable information for policy making and a reference for antenatal depression screening and interventions in comprehensive hospitals to improve maternal and neonatal outcomes.

## Methods

### Study design and population

A Cross-sectional study was conducted in Chongqing, a major city almost with thirty-one million residents, thirteen million female and 32,000 pregnant women, southwest of China. All the women diagnosed with pregnancy by B-ultrasonography and registered to attending antenatal care at the Department of Obstetrics, the First Affiliated Hospital of Chongqing Medical University were required to complete online psychological assessments for free at their first attending antenatal care, but some pregnant women who insisted on refusing psychological assessment or were illiterate were excluded. The subjects were informed about the purpose of the online psychological assessments, the confidentiality of personal information and the principle of volunteering. Written informed consent was obtained from all participants before evaluation. Considering the impact of COVID-19 outbreak on maternal mental health, only data during June 1 to December 31, 2019 were included for analysis in the study.

### Data collection

The survey was conducted online via QR code and website specifically designed for the present study. Access to the online questionnaires was restricted to pregnant women enrolled in the study using the unique telephone number for login. These assessments began with an overall introduction about the purpose of the project, and then moved on to online instructions for each specific questionnaire. Psychological assessments were composed of two parts: general information and psychiatric symptoms assessments. Identified assessment instructions were provided before each assessment scale.. All participants completed the online psychological assessments by themselves in a quiet room and two trained nurses were on hand to answer any questions they had. After the evaluation submitted, the nurse reviewed the scale scores and wrote them down on the antenatel record.

### Depression, anxiety, and somatization symptoms assessment

The Patient Health Questionaire-9 (PHQ-9) is a 9-item scale to assess depression symptoms in the 14 days prior to assessment, assigning scores of 0 ~ 3 according to the response categories (0=“none at all”, 1 = “several days”, 2 =“more than half the days”, and 3 =“nearly every day”). The results categories were as follows: 0 ~ 4 were classified as normal, 5 ~ 9 as mild depression, 10 ~ 14 as moderate depression, 15 ~ 19 as moderate to severe depression, and 20 ~ 27 as severe depression. Depression was defined as score of 10 or higher, and for this cut-off point, the sensitivity and specificity were 88 and 86%, respectively [19]. In this study, the Cronbach’s alpha of the PHQ-9 was 0.87.

The severity of anxiety was assessed using the Chinese-language version of Generalized Anxiety Disorder-7 in the 14 days prior to evaluation [20]. The GAD-7 score was calculated with scores of 0 ~ 3, corresponding to the following response options: 0=“not at all”, 1 = “several days”, 2=“more than half the days” and 3=“nearly every day”. The results categories were as follows: 0 ~ 4 were classified as normal, 5 ~ 9 as mild anxiety, 10 ~ 14 as moderate anxiety, and 15 ~ 21 as severe anxiety. Anxiety was defined as a cut-off score of 10 or higher, with a sensitivity of 86.2% and specificity of 95.5% [21]. In this study, the Cronbach’s alpha of the GAD-7 was 0.89.

The Patient Health Questionnaire-15 (PHQ-15) was used to assess the number and severity of somatic symptoms in the past 4 weeks prior to the evaluation. There were a total of 15 somatic symptom items, scored from 0 ~ 2 to the following response: 0 = “no disturbance”, 1 = “little disturbance” and 2 = “much disturbance”. The results criteria: 0 ~ 4 were classified as normal, 5 ~ 9 as mild somatic symptoms, 10 ~ 14 as moderate somatic symptoms, and 15 ~ 30 as severe somatic symptoms. The Chinese version of the PHQ-15 has good reliability and validity in the general population [22]. A cut-off point of 10 or higher was used to assess the presence of somatic symptoms, with a sensitivity of 80.2% and a specificity of 58.5% [23]. In this study, the Cronbach’s alpha of PHQ-15 was 0.85.

### Variables

The main outcome was depression defined by PHQ-9 ≥ 10. Participants’ age was categorized as follows: ≤ 24, 25–29, 30–34, 35–39, or ≥ 40 years old. Other sociodemographic variables were nationality (Han vs other), residence (rural vs urban), marital status (married, unmarried or divorced, and remarried), level of education (middle school or lower, high school, college, master’s degree or higher), parity (nulliparous vs multiparous), occupation (fixed employed, self-employed, unemployed) and gestational weeks at interview.

### Quality control

All entries were set as compulsory questions and the IP address verified by the mobile phone of the tester could only save final answer on the test day. The questionnaires would only be submitted after completing all items. Otherwise, the system would automatically identify the outcome as incomplete. Pretest were carried out to check the suitability and understandability of the questionnaires and functionality of the website. Online psychological assessments had been adopted to adolescent depression or suicide screening in in our groups previous researches [24]. The test time was set by pretest results, and questionnaires with a test duration of less than 180 s were deleted.

The study has been approved by by the ethics committee of Chongqing Medical University, China.

### Statistical analysis

Data were analysed in IBM SPSS Statistics 22.0. Descriptive statistics was applied to the study variables and general characteristics. We compared demographic variables between pregnant women with and without depression by using the chi-square test for categorical variables and the Mann-Whitney nonparametric test or ANOVA for nonnormally or normally distributed continuous variables, respectively. Furthermore, we applied binary logistic regression analysis to examine the factors related to antenatal depression. Odds ratios (ORs) with their 95% confidence intervals (*CIs*) were calculated to measure the strength of association. *P* (two-tailed) <  0.05 was considered statistically significant.

## Results

### Subject characteristics

During June 1 to December 31, 2019, a total of 6148 pregnant women finished registration for antenatal care. Of which 368 pregnant women didn’t attend the online psychological assessment (these participants refused psychological assessment or were illiterate). Thus, 5780 pregnant women attended the online psychological questionnaire survey. Fifty-two women were excluded due to being postpartum or missing information on any of the three scales (PHQ-9, GAD-7, PHQ-15). A total of 5728 women remained for the analysis. Hence, the effective rate of valid data received for the present analysis was 99.1%.

Table 1 shows the general sociodemographic characteristics and associated factors of the participants enrolled in our study. Most participants were aged between 25 and 34 years (*n* = 4190; 73.1%), were urban residents (*n* = 3108; 54.3%), were of Han nationality (*n* = 5407; 94.4%), were married (*n* = 4984; 87%), were employed (*n* = 4629; 80.8%), and had at least a high school education (*n* = 90%). A total of 3283 (57.3%) participants were multigravida. The proportions of pregnant women in the first, second and third trimesters were 73, 18, and 9%, respectively. A total of 16.3% (*n* = 933) of pregnant women had depression (PHQ-9 ≥ 10), which was higher than the proportion of the healthy Chinese population with depression [2]. In addition, 25.7% (*n* = 1471) of pregnant women had significant somatic symptoms (PHQ-15 ≥ 10), and 4.4% (*n* = 252) of pregnant women had significant anxiety (GAD≥10). Compared to anxiety and depression, somatic symptoms were more common for women during pregnancy.

**Table 1:** Demographic characteristics and univariate analysis showing factors related with antenatal depression

Characteristic	Total sample (*n* = 5728)	PHQ-9 ≥ 10 (*n* = 933)	PHQ-9 < 10 (*n* = 4795)	x^2^/F	*P*
Age, No. (%)				12.506	0.014*
≤ 24	654 (11.4)	134 (14.4)	520 (10.8)		
25–29	2322 (40.5)	366 (39.2)	1956 (40.8)		
30–34	1868 (32.6)	288 (30.9)	1580 (33.0)		
35–39	575 (10.0)	86 (9.2)	489 (10.2)		
≥ 40	309 (5.4)	59 (6.3)	250 (5.2)		
Residence, No. (%)				29.989	< 0.001*
Rural	2620 (45.7)	503 (53.9)	2117 (44.2)		
Urban	3108 (54.3)	430 (46.1)	2678 (55.8)		
Race, No. (%)				0.538	0.46
Han nationality	5407 (94.4)	876 (93.9)	4531 (94.5)		
Others	321 (5.6)	57 (6.1)	264 (5.5)		
Marital status, No. (%)				11.599	0.003*
Married	4984 (87.0)	781 (83.7)	4203 (87.7)		
Unmarried/divorce)	674 (11.8)	135 (14.5)	539 (11.2)		
Remarried	70 (1.2)	17 (1.8)	53 (1.1)		
Education, No. (%)				29.689	< 0.001*
Middle school or less	570 (10.0)	114 (12.2)	456 (9.5)		
High school	818 (14.3)	156 (16.7)	662 (13.8)		
Technical secondary school	1770 (30.9)	314 (33.7)	1456 (30.4)		
College	2171 (37.9)	306 (32.8)	1865 (38.9)		
Master or higher	399 (7.0)	43 (4.6)	356 (7.4)		
Gravidity, No. (%)				3.337	0.068
Primigravida	2445 (42.7)	373 (40.0)	2072 (43.2)		
Multigravida	3283 (57.3)	560 (60.0)	2723 (56.8)		
Occupation, No. (%)				1.714	0.424
Fixed employed	3782 (66.0)	612 (65.6)	3170 (66.1)		
Self-employed	847 (14.8)	150 (16.1)	697 (14.5)		
Not employed	1099 (19.2)	171 (18.3)	928 (19.4)		
Gestational weeks, No. (%)				42.802	< 0.001*
First trimester (< 14)	4179 (73.0)	755 (80.9)	3424 (71.4)		
Second trimester (14–28)	1035 (18.0)	137 (14.7)	898 (18.7)		
Third trimester >2)	514 (9.0)	41 (4.4)	473 (9.9)		
PHQ-15 score, X ®±S	6.85 ± 4.34	11.04 ± 4.42	6.03 ± 3.82	−29.672	< 0.001*
GAD-7 score, X ®±S	2.96 ± 3.23	6.44 ± 4.53	2.28 ± 2.38	− 29.896	< 0.001*

For continuous variables, *P*-value was calculated using the ANOVA for normally distributed or the Mann-Whitney nonparametric test for nonnormally distributed; For categorical variables, *P*-value was calculated using the chi-square test.

*Statistically significant: *P* <  0.05

Table 1 also shows that gestational age (*p* = 0.014), residence (*p* <  0.001), marital status (*p* = 0.003), educational level (*p* <  0.001) and gestational weeks (*p* <  0.001) might be related with antenatal depression for women, and difference between the two groups had statistical significance (*p* < 0.05). Additionally, both PHQ-15 scores (*p* <  0.001) and GAD-7 scores (*p* <  0.001) of pregnant women with depression were significantly higher than those of pregnant women without depression (see Table 1).

To further clarify the relationship among antenatal depression, anxiety and somatic symptoms, the risk of antenatal depression among pregnant women with different levels of anxiety or somatic symptoms was compared. Table 2 shows that the severity of anxiety or somatic symptoms was significantly associated with the risk of antenatal depression for women (*p* <  0.001).

**Table 2:** Relation among GAD level, PHQ-15 level and antenatal depression

Characteristics	Total sample (*n* = 5728)	PHQ-9 ≥ 10 (*n* = 933)	PHQ-9 < 10 (*n* = 4795)	*X*^*2*^	*P*
GAD level, No. (%)				1173.408	< 0.001*
No anxiety symptoms	4396 (76.7)	368 (39.4)	4028 (84.0)		
Mild anxiety	1080 (18.9)	367 (39.3)	713 (14.9)		
Moderate anxiety	154 (2.7)	114 (12.2)	40 (0.8)		
Moderate-severe anxiety	77 (1.3)	65 (7.0)	12 (0.3)		
Severe anxiety	21 (0.4)	19 (2.0)	2 (0.0)		
PHQ-15 level, No. (%)				1021.353	< 0.001*
No somatic symptoms	1905 (33.3)	52 (5.6)	1853 (38.6)		
Mild somatic symptoms	2352 (41.1)	300 (32.2)	2052 (42.8)		
Moderate somatic symptoms	1165 (20.3)	387 (41.5)	778 (16.2)		
Severe somatic symptoms	306 (5.3)	194 (20.8)	112 (2.3)		

For categorical variables, *P*-value was calculated using chi-square test.

*Statistically significant: *P* < 0.05

Furthermore, binary logistic regression was used to determine the relationship between antenatal depression and its associated factors. All the factors with significant differences (*P <* 0.05) by univariate analysis (see Table 1) were included in binary regression analysis. Only residence, somatic symptoms level (PHQ-15 score),anxiety level (GAD-7 score) and and trimesters of pregnancy were found associated with antenatal depression and remained statistically significant (*P <* 0.05) afer adjusting for age, educational level, marital status, race, gravidity (see Table 3). Table 3 also shows that the protective factors against antenatal depression were gestational weeks and urban residence (AOR = 0.786; 95% CI: 0.652–0.947). Compared with the first trimester, the risk of depression was lower in the second (AOR = 0.611; 95% CI: 0.483–0.773), and third trimesters (AOR = 0.337; 95% CI: 0.228–0.498). It was also found that anxiety symptoms and somatic symptoms were risk factors for antenatal depression. Compared with pregnant women without somatic or anxiety symptoms, women with some degree of anxiety or somatic symptoms were at least 2.937 times (Mild anxiety AOR = 2.937; 95% CI: 2.448–3.524) or 3.938 times (Mild somatic symptoms AOR = 3.938; 95% CI: 2.888–3.368) more likely to develop depression. Moreover, the higher the level of anxiety or somatic symptoms was the greater the risk of developing depression was.

**Table 3:** Binary logistic analysis significant factors associated with antenatal depression

Variables		B	SE	Wald	*P*	OR	95% CI	
Gestational weeks	First trimester (< 14)			42.247	0.000^**^			
	Second trimester (14–28)	−0.493	0.120	16.813	0.000^**^	0.611	0.483	0.773
	Third trimester (>28)	−1.088	0.199	29.886	0.000^**^	0.337	0.228	0.498
Urban residence		−0.241	0.095	6.411	0.011	0.786	0.652	0.947
Anxiety level	No anxiety symptoms			317.947	0.000^**^			
	Mild anxiety	1.077	0.093	134.333	0.000^**^	2.937	2.448	3.524
	Moderate anxiety	2.666	0.209	163.046	0.000^**^	14.387	9.555	21.663
	Moderate-severe anxiety	3.155	0.345	83.625	0.000^**^	23.45	11.925	46.110
	Severe anxiety	4.125	0.837	24.309	0.000^**^	61.897	12.007	319.086
Somatic symptoms level	No somatic symptoms			310.108	0.000^**^			
	Mild somatic symptoms	1.371	0.158	75.085	0.000^**^	3.938	2.888	5.368
	Moderate somatic symptoms	2.283	0.163	196.955	0.000^**^	9.804	7.128	13.485
	Severe somatic symptoms	3.062	0.202	230.121	0.000^**^	21.38	14.394	31.758

Only the above four factors remained statistically significant (**: *P* < 0.001) after adjusting for age, education level, marital status, race, gravidity.

*Abbreviations*: *PHQ-15* patient health questionnaire-15; *GAD-7* Generalized anxiety disorder; *CI* confidence interval

## Discussion

To the best of our knowledge, this was the first study in China in all pregnant women regardless of whether they had severe physical or mental disease. The prevalence of antenatal depression at their first attending antenatal care in our study was 16.3%, which was equivalent to that reported in a recent meta-analysis [5], but relatively lower than estimates reported in most previous studies in China. For example, Li et al. reported the prevalence of antenatal depression evaluated by the Edinburgh Postpartum Depression Scale (EPDS) was 19.1% [11]. And Chen et al. found that the prevalence of antenatal depression was 29.6% [15], but in this study, the cut-off point for depression on the EPDS was 9, lower than that in the Li’s study. The tools used to assess depression [25] and the inconsistent cut-off point for the EDPS used in previous studies can partly explain the disparities. Nine [15], 10 [17], 12 [11], or 13 [26] have been chosen as cut-off points for depression on the EDPS. In addition, the study population might cause these differences. In our study, all pregnant women were enrolled regardless of whether the pregnant woman had a serious physical or mental illness. Evidence showed pregnant women with serious physical and mental disease had a higher incidence of antenatal depression. For example, the prevalence of antenatal depression in HIV-positive pregnant women is higher [27, 28] and a history of mental health problems increases the risk of developing antenatal depression [29]. Third, gestational weeks might affect the prevalence of antenatal depression, and a study reported that the prevalence of depression varied in the three trimesters during pregnancy [5]. This study was a real-world study among all pregnant women. Therefore, the results of our study were more likely to reflect the real status of prenatal depression in the obstetric clinics in the comprehensive teaching hospital setting.

The prevalence of antenatal depression in our study was significantly lower than that reported in low- and middle-income areas, such as Africa (26.3%) [6], but slightly higher than that reported in high-income countries, such as Ireland [30]. This discrepancy in prevalence between countries might be due to the differences in depression screening tool, culture, study methodology, study population, socio- economic and socio- demographic variations. In a word, depression in pregnant women has become a public health problem, and more attention should be given to its prevention and intervention.

We also found that significant risk factors for depression among pregnant women were anxiety level, somatic symptom level, rural residence and early pregnancy. Strong evidence has confirmed that depression is closely related to anxiety and somatic symptoms [31]. For example, Patients with somatic discomfort disorder [32, 33] or anxiety disorder [34, 35] are at greater risk of depression, and patients with depression usually usually have anxiety and somatic symptoms. Consistent with our research, Barthel et al. also found that anxiety was a risk factor for perinatal depression [36] and Apter et al. reported that the total score for somatic complaints, as evaluated by a checklist of 18 somatic complaints, moved from 3 to 7, and the risk of antenatal depression increased 1.91 times [37]. Another study reported that changes in energy levels were responsible for the increased prevalence of depression in pregnant women [38]. As a whole, there have been few studies on the association between antenatal depression and somatic symptoms or anxiety.

Somatic symptoms (nausea and vomiting or morning sickness) are very common complaints during early pregnancy, affecting almost 60 ~ 80% of pregnant women [39, 40]. Severe or long duration of somatic symptoms might bring distress or stress to pregnant women and increase the risk of depression. In addition, inflammation and oxidative stress might also lead to the higher prevalence of depression in women with somatic symptoms during pregnancy. Inflammation is one of the important pathophysiological processes of depression [41–44]. Eutrophil-to-lymphocyte ratio (NLR), platelet-to-lymphocyte ratio (PLR) and CRP are clinical common indicators used to reflect the inflammatory response. NLR and PLR of hyperemesis gravidarum patients were significantly higher than the healthy pregnant women [45, 46]. Consistent findings were also found in other inflammatory markers, such as white blood cell count, total oxidative status (TOS) [47], CRP [48, 49]. So, we speculate somatic symptoms during pregnancy might induce higher inflammation and oxidative stress level, which in turn lead to higher prevalence of depression.

Our study also found that gestational weeks was associated with antenatal depression, with a higher incidence in early pregnancy than in the second or third trimester. Combined with the correlation between antenatal depression and somatic symptoms found in this study. We speculated that somatic symptoms associated with early pregnancy, such as nausea, vomiting, frequent urination, fatigue and constipation, might be responsible for the higher prevalence of depression in early pregnancy. In addition, another important factor might be spontaneous abortion, the most common complication in the first trimester of pregnancy. And its incidence decreases with the increase of gestational weeks. Zhu et al. reported that women facing threatened miscarriage had a higher risk for major depressive and anxiety symptoms than those with stable pregnancies [50]. Al-Memar et al. found that first-trimester vaginal bleeding was an independent risk factor for adverse obstetric outcomes [51], which might increase the risk of antenatal depression [52] and were also significant predictors of antenatal depression (adjusted OR 3.14) [53]. However, further studies are needed to determine the causes of the differences in the prevalence of depression during pregnancy and the interaction among antenatal depression, anxiety and somatic symptoms. Therefore, More attention should be paid to the pregnant women with morning sickness and vaginal bleeding in the first trimester and timely explanation, mental health evaluation, counselling and psychological support should be given to reducing the risk of antenatal depression.

Moreover, this study also found that rural residence was risk factor for antenatal depression, which might be indirectly caused by low income. According to China’s National Bureau of Statistics, the per capita disposable income of urban residents in 2019 was 42,359 yuan, while that of rural residents was only 16,021 yuan, much lower than that of urban residents. Accordingly, Studies have reported that low income and financial stress are risk factors for antenatal depression [6]. Therefore, pregnant women with low income level, especially from rural areas, should be given more attention to mental health.

There are still some limitations in this study. It was a cross-sectional study, so the relationship between variables could not be proven. And other psychosocial factors that might affect depression in pregnant women was not evaluated, such as marital relationship, financial income, history of childhood trauma, relationship between mother-in-law and daughter-in-law and partner violence. In addition, Our findings actually might be more representative of early pregnancy depression, because pregnant women in early pregnancy accounted for the majority of subjects. A longitudinal study might be more helpful in clarifying the status of antenatal depression. Moreover, all pregnant women were enrolled in the study and anomaly screening tests, important factors that may predispose women to depression, were not distinguished between participants and the inclusion of the entire population, a large sample might also increase the rate of false discovery. But, as mentioned above, the main purpose of this study was to present the real status of antenatal depression in the obstetrics clinic. And the associated risk factors we found also have been replicated in other studies, so the sample size did not affect the reliability of the results. In light of the above limitations, further studies would be conducted to investigate more psychosocial factors and dynamically assess depression in women during different pregnancy stages.

## Conclusion

Almost one in six pregnant women might have prenatal depression and strengthening the screening and early intervention of depression during pregnancy is critical. Online mental health assessment might be a time-saving and convenient screening method for depression and should be widely used for pregnant women in obstetric clinics, especially for women with certain risk factors (anxiety symptoms, somatic symptoms, rural residence and early pregnancy) .

## Data Availability

All data included in this study are available during the current study are available from the corresponding author on reasonable request.

## Abbreviations

PHQ-9: Patient health questionnaire-9
GAD-7: Generalized anxiety disorder-7
PHQ-15: Patient health questionnaire-15
EPDS: Edinburgh postnatal depression scale
MDT: Multidisciplinary team
WHO: World health organization
TOS: Total oxidative status
NLR: Neutrophil-to-lymphocyte ratio
PLR: Platelet-to-lymphocyte ratio
CRP: C-reactive protein
DSM: Diagnostic and statistical manual of mental disorders
AOR: Adjusted OR
